# PACAP Interacts with PAC_1_ Receptors to Induce Tissue Plasminogen Activator (tPA) Expression and Activity in Schwann Cell-Like Cultures

**DOI:** 10.1371/journal.pone.0117799

**Published:** 2015-02-06

**Authors:** Alessandro Castorina, James A. Waschek, Rubina Marzagalli, Venera Cardile, Filippo Drago

**Affiliations:** 1 Department of Biomedical Sciences and Biotechnologies, Section of Human Anatomy and Histology, University of Catania, Catania, Italy; 2 Semel Institute/Department of Psychiatry, David Geffen School of Medicine, University of California Los Angeles, Los Angeles, California, United States of America; 3 Department of Biomedical Sciences and Biotechnologies, Section of Physiology, University of Catania, Catania, Italy; 4 Department of Biomedical Sciences and Biotechnologies, Section of Pharmacology, University of Catania, Catania, Italy; University of Rouen, FRANCE

## Abstract

Regeneration of peripheral nerves depends on the abilities of rejuvenating axons to migrate at the injury site through cellular debris and altered extracellular matrix, and then grow along the residual distal nerve sheath conduit and reinnervate synaptic targets. Considerable evidence suggest that glial cells participate in this process, although the mechanisms remain to be clarified. In cell culture, regenerating neurites secrete PACAP, a peptide shown to induce the expression of the protease tissue plasminogen activator (tPA) in neural cell types. In the present studies, we tested the hypothesis that PACAP can stimulate peripheral glial cells to produce tPA. More specifically, we addressed whether or not PACAP promoted the expression and activity of tPA in the Schwann cell line RT4-D6P2T, which shares biochemical and physical properties with Schwann cells. We found that PACAP dose- and time-dependently stimulated tPA expression both at the mRNA and protein level. Such effect was mimicked by maxadilan, a potent PAC1 receptor agonist, but not by the PACAP-related homolog VIP, suggesting a PAC1-mediated function. These actions appeared to be mediated at least in part by the Akt/CREB signaling cascade because wortmannin, a PI3K inhibitor, prevented peptide-driven CREB phosphorylation and tPA increase. Interestingly, treatment with BDNF mimicked PACAP actions on tPA, but acted through both the Akt and MAPK signaling pathways, while causing a robust increase in PACAP and PAC1 expression. PACAP6-38 totally blocked PACAP-driven tPA expression and in part hampered BDNF-mediated effects. We conclude that PACAP, acting through PAC1 receptors, stimulates tPA expression and activity in a Akt/CREB-dependent manner to promote proteolytic activity in Schwann-cell like cultures.

## Introduction

One of the major challenges regenerating neurites have to cope with after peripheral nerve injury is to migrate through cellular debris and the altered extracellular matrix (ECM) at the injury site, grow along the residual distal nerve sheath conduit, and reinnervate synaptic targets [3]. To achieve this goal, growth cone of regenerating axons secrete proteases capable of degrading matrix molecules and cell adhesions.

Tissue plasminogen activator (tPA) is a serine protease that cleaves the proenzyme plasminogen to its active form, plasmin. Plasmin exerts proteolytic activity on a broad spectrum of substrates including most ECM molecules and cell-adhesion molecules [16]. Additionally, plasmin can act indirectly thorough the activation of several other matrix metalloproteases (MMPs) to complement its degrading activities [30]. tPA has also demonstrated some plasminogen-independent proteolytic activities, including the activation of a neuronal responsive growth factors [27], as well as the cleavage of fibronectin, a component of the nervous scar tissue that impedes normal axonal regeneration after injury [20, 28].

A number of investigations have documented the presence of plasminogen activators (PAs) in neurons and PA system involvement in axonal outgrowth. PAs are secreted by cultured peripheral neurons, but previous studies have indicated that they are also released by Schwann cells [24]. However, the molecular switch that drives tPA induction and activity in either cellular populations has not received much attention.

PACAP as well as the structurally-related peptide vasoactive intestinal peptide (VIP) belong to a family that includes secretin, glucagon and peptide histidine-isoleucine (PHI), which are involved in a plethora of biological functions [15, 44]. PACAP and VIP effects are mediated by three different G-protein-coupled receptors, the PAC_1_ type (with at least eight known splice variants) and the VPAC type (including VPAC_1_ and VPAC_2_ receptor subtypes). PAC_1_ receptors bind with higher affinity PACAP than VIP. Both VPAC_1_ and VPAC_2_ receptors bind similarly and with high affinity PACAP and VIP. Each of these receptors displays seven transmembrane domains and activates primarily cAMP, but also other transduction systems [23, 29, 50].

Despite the number of biological functions already described for PACAP and VIP, new interesting and emerging role for both peptides are still arising. Indeed, Raoult and coworkers, (2011) [35] have recently demonstrated that PACAP is a potent inducer of tPA expression in both tumoral and normal neuronal cells, suggesting a novel mechanism of action for the peptide in nerve repair after injury. Nonetheless, Schwann cells also release PAs to aid nerve regeneration, but whether or not PACAP contributes to this process is still unknown, nor are the signaling pathways involved.

In this regard, the present study aimed at investigating whether the neuropeptide PACAP promoted the expression and activity of tPA in the RT4-D6P2T schwannoma cell line, a Schwann cell-like culture system endowed with biochemical and structural features of normal myelinating Schwann cells [10, 11, 21, 37].

Throughout our investigations we revealed that, similarly to neurons, PACAP potently stimulated tPA expression and activity in RT4-D6P2T cells. Furthermore, we also determined that PAC_1_ receptors mediated the activation of the Akt/CREB signaling pathway to induce tPA. Finally, we showed that brain derived neutrophic factor (BDNF) effectively mimicked PACAP in stimulating tPA expression through a mechanism involving simultaneous activation of both the MAPK- and Akt-CREB signaling pathways, and indirectly by upgrading the endogenous PACAP/PAC_1_ system by increasing their expression levels.

## Materials and Methods

### Cell culture

The present study was performed using the rat Schwann cell-like culture RT4-D6P2T (ATCC number CRL-2768) obtained from the American Type Culture Collection (Rockville, MD, USA).

Cells were cultured in Dulbecco’s modified Eagle’s medium (DMEM) and supplemented with 10% of heat-inactivated fetal bovine serum (FBS), 100U/ml penicillin, and 100 μg/ml streptomycin (Lonza, Italy). Cells were incubated at 37°C in a humidified atmosphere with 5% CO_2_. Cells were grown to reach about 80–85% confluence in media containing 10% FBS and subjected to different treatments as described in the related subsections.

### Western blot analysis

Western blot analysis was performed according to the procedures previously described by Giunta et al., (2010) [17]. Briefly, proteins were extracted using an ice-cold lysis buffer containing 20mM Tris (pH 7.4), 2mM EDTA, 0.5mM EGTA; 50mM mercaptoethanol, 0.32mM sucrose, a protease inhibitor cocktail and the phosphatase inhibitor PhosSTOP (Roche Diagnostics) using a Teflon-glass homogenizer and then sonicated twice for 20s using an ultrasonic probe, followed by centrifugation at 10.000g for 10min at 4°C. Protein concentrations were determined using the Quant-iT Protein Assay Kit (Invitrogen, Carlsbad, CA, USA). Sample proteins (20–35μg) were diluted in 2X Laemmli buffer (Invitrogen), heated at 70°C for 10 min and then separated on a Biorad Criterion XT 4–15% Bis-tris gel (Invitrogen) by electrophoresis and then transferred to a nitrocellulose membrane (Invitrogen). Blots were blocked using the Odyssey Blocking Buffer (Li-Cor Biosciences). Effective transfer was monitored using a prestained protein molecular weight marker (BioRad Laboratories). Immunoblot analyses were performed using the following primary antibodies: rabbit anti-tPA (1:300, sc-15346, Santa Cruz Biotechnology), mouse anti-phospho Erk-1/2^(Thr202-Tyr204)^ (pT202/pY204.22A, cat n. sc-136521, Santa Cruz Biotechnology; 1:200), mouse anti-total Erk-1/2 (MK1, cat n. sc-135900, Santa Cruz Biotechnology; 1:200), rabbit anti-phospho Akt^(Ser473)^ (D9E, cat n. #4060, Cell Signaling; 1:1000), rabbit anti-total Akt (C67E7, cat n. #4691, Cell Signaling; 1:1000), phospho-CREB^(Ser133)^ (1:300, sc-7978, Santa Cruz Biotechnology), total CREB (1:300, sc-186, Santa Cruz Biotechnology) and a rabbit anti-β-tubulin (H-235, cat n. sc-9104, Santa Cruz Biotechnology; 1:500).

The secondary antibodies goat anti-rabbit IRDye 800CW, (cat #926-32211; Li-Cor Biosciences) and goat anti-mouse IRDye 680CW, (cat #926-68020D; Li-Cor Biosciences) were used at the dilution of 1:20000 and 1:30000 respectively. Blots were scanned using an Odyssey Infrared Imaging System (Li-Cor Biosciences). Densitometric analyses of bands were performed at non-saturating exposures using the ImageJ software (NIH, Bethesda, MD; available at http://rsb.info.nih.gov/ij/). Values of non-phosphorylated proteins were normalized to β-tubulin, which served as loading control, whereas values from phosphorylated proteins were normalized to their respective unphosphorylated total protein levels. Background correction values were subtracted from each lane to minimize the variability across membranes. No signals were detected when primary antibodies were omitted (data not shown).

### Quantitative real time polymerase chain reaction

Total RNAs from RT4-D6P2T cells exposed to different treatments were isolated using 1 ml TRIzol reagent (Invitrogen) and 0.2 ml chloroform and precipitated with 0.5 ml isopropanol. Pellets were washed with 75% ethanol and air dried. Single stranded cDNAs were synthesized by incubating total RNA (5μg) with SuperScript III RNase H-reverse transcriptase (200 U/μl) (Invitrogen); Oligo-(dT)_20_ primer (100 nM) (Invitrogen); 1 mM dNTP mix (Invitrogen), dithiothreitol (DTT, 0.1 M), recombinant RNase-inhibitor (40 U/μl) at 42°C for 1 h in a final volume of 20μl. Reaction was terminated by incubation of samples at 70°C for 10 min.

Aliquots of cDNA (100ng) from each sample and external standards at known amounts (purified PCR products, ranging from 10^2^ to 10^8^ copies) were amplified in parallel reactions, using procedures previously described in our lab [18]. Briefly, primer pairs were designed to specifically recognize tissue plasminogen activator, forward 5’- GCTCCCTGACTGGACAGAGT-3’ and reverse 3’-CGGCTGGACGGATACAGTCT-5’ (Acc# NM_013151.2) and the S18 ribosomal subunit, forward 5’-CCTGCGAGTACTCAACACCA-3’ and reverse 3’-CTGCTTTCCTCAACACCACA-5’ (Acc# NM_213557.1), which was measured in each amplification and used as the reference gene. Each PCR reaction contained 0.5 μM primers, 1.6 mM MgCl^2+^, 1X Light Cycler-FastStart DNA Master SYBR Green I (Roche Diagnostic). Amplifications were performed using the Light Cycler 1.5 instrument (Roche Diagnostic) using the following program setting: (I) cDNA denaturation (1 cycle: 95°C for 10 min); (II) quantification (45 cycles: 95°C for 10 s, 60°C for 30 s, 72°C for 7 s); (III) melting curve analysis (1 cycle: 95°C for 0 s, 65°C for 15 s, 95°C for 0 s); (IV) cooling (1 cycle: 40°C for 30 s). Quantification was obtained by comparing the fluorescence emitted by PCR products at unknown concentration with the fluorescence emitted by external standards at known concentration. For this analysis, fluorescence values, measured in the log-linear phase of amplification, were estimated with the second derivative maximum method using Light Cycler Data Analysis software. PCR products specificity was evaluated by melting curve analysis.

To assess the different expression levels we analyzed the mean fold change values of each sample, calculated using the comparative Ct method [38]. ΔCt was calculated by normalizing the mean Ct of each sample to the mean Ct of the reference gene measured in the same experimental conditions. For the quantification of each gene we considered cDNAs from untreated RT4-D6P2T cells as the calibrator sample. The ΔΔCt of each sample was then calculated by subtracting calibrator ΔCt to treated sample ΔCt. The formula 2^−ΔΔCt^ was used to calculate fold changes. Baseline measurements for each calibrator sample were set to 1.

### Immunofluorescence microscopy

Rat RT4-D6P2T Schwann cell-like cells cultured on glass cover slips were fixed in 4% para-formaldehyde (PFA) in PBS (15′ at room temperature), permeabilized with 0.2% Triton X-100, blocked with 0.1% BSA in PBS, then probed with the rabbit anti-tPA primary antibody (1:50). Immunodetection was then performed on cells cultured on glass cover slips using the Alexa fluor 488-conjugated secondary antibody raised against rabbit, with an exposure time of 1.5–2h at room temperature and shielded from light. DNA was counterstained with DAPI (#940110 Vector Laboratories). After a series of PBS and double-distilled water washes, fixed cells were cover-slipped with the Vectashield mounting medium (Vector Laboratories, Inc., Burlingame, CA).

### Image acquisition and processing

Immunofluorescent images were acquired using an Axiovert 40 CFL inverted fluorescence microscope (Carl Zeiss Inc.) equipped with an AxioCam MRc5 digital color camera (Carl Zeiss Inc.). Each condition was reproduced in three dishes *per* experiment. Representative photomicrographs were taken from at least three fields *per* dish in a fixed pattern. All microscope settings were set to collect images below saturation and were kept constant for every image taken in one experiment. All images were collected at 16-bit resolution in order to maximize the dynamic range of the detected intensities. Relative fluorescence was determined using the open source software ImageJ from the NIH (http://rsbweb.nih.gov/ij/). Nuclei were defined using the DAPI channel (435–485 nm emission) using a 420nm longpass filter, whereas tPA immunosignals were defined using the Fluorescein Iso-thiocyanate (FITC) channel and collected through a 525/50nm bandpass filter. To avoid the possibility of DAPI spectral bleedthrough into Alexa fluor 488 dye, fluorescent signals were captured and recorded on separate channels using sequential imaging and merged afterwards. Regions of Interest (ROIs) used to quantify the integrated intensity of fluorescent images were selected by applying a macro with preset threshold level, gamma, brightness and contrast values, which were kept constant throughout the experiments. Background values were extrapolated from at least five separate fixed areas taken from the same photomicrograph. To calculate relative fluorescence intensity, expressed as mean values in arbitrary fluorescence units (a.u.), we applied the following formula:

Relative fluorescence = Integrated density of stained area-(mean background area * mean fluorescence of background readings)

Zymography of tissue type plasminogen activator activity

Following treatment with 2x10^−9^M BDNF or 10^−7^M PACAP, cells were collected with 1% Triton X-100 in PBS buffer. The activities of tissue- and urokinase-type plasminogen activator (tPA and uPA, respectively) were determined by zymographic analysis. Equal amounts of protein were loaded onto 12% polyacrylamide gel containing 2 mg/ml casein in the presence of 5 μg/ml plasminogen. After electrophoresis, enzyme reaction was initiated by incubating the gel in 0.1 M glycine-NaOH (pH 8.3) at 37°C for 18 h, and lytic areas were identified after staining of the gel with a solution containing 30% methanol, 10% glacial acetic acid, and 0.5% Coomassie blue G250. The gels were then destained in the same solution in the absence of the dye and scanned with Bio-Rad Imaging system. Images were assessed semiquantitatively using the ImageJ software. To exclude interference by matrix metalloproteinases (MMPs), EDTA (2 mM) was included in the glycine-NaOH buffer during the incubation period. Gels that did not containing plasminogen were also run and failed to produce lytic areas corresponding to plasminogen activators (data not shown). Bands were identified based on their relative molecular weight.

### Statistical analysis

Statistical analysis was performed using GraphPad Prism version 4.00 for Windows, GraphPad Software, San Diego California USA, www.graphpad.com. All experimental data are reported as mean ± S.E.M. To assess statistical differences between three or more groups we used one-way analysis of variance (ANOVA) followed by Tukey *post-hoc* test, unless otherwise stated. *p* values ≤ 0.05 were considered statistically significant.

## Results

### PACAP38 dose- and time-dependently increases tPA mRNA and protein expression in RT4-D6P2T cells

**Fig. 1:**
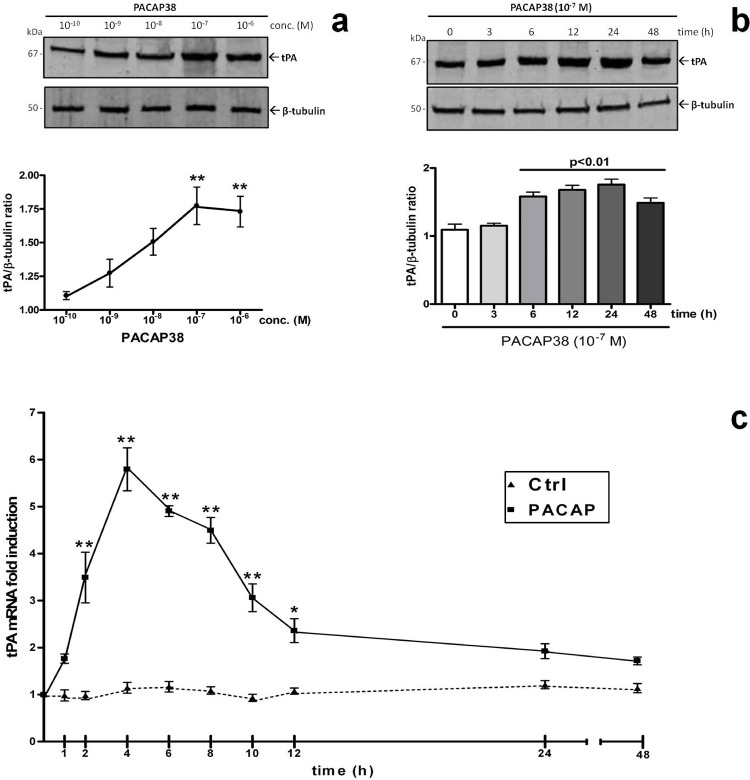
Dose- and time-dependent effects of PACAP on tissue plasminogen activator (tPA) mRNA and protein expression. Representative immunoblots and densitometric analyses (**a – b**) showing the effects of increasing concentrations of PACAP38 (10^−10^M to 10^−6^M, respectively) or (**a**) of a single concentration of the peptide (10^−7^M) on tPA protein expression at different time points (0, 3, 6, 12, 24, 48h). Protein extracts (20μg) obtained from rat RT4-D6P2T cultures grown as described were separated by SDS-PAGE and transferred to nitrocellulose membranes. Then, membranes were incubated using a rabbit anti-tPA (1:300, sc-15346, Santa Cruz Biotechnology) and a rabbit anti-β-tubulin antibody (H-235, cat n. sc-9104, Santa Cruz Biotechnology; 1:500) and scanned with an Odyssey Infrared Imaging System, as described in Materials and Methods section. Densitometric analyses were performed using the ImageJ software and values obtained were normalized to β-tubulin, which was used as loading control. Results are expressed as the average ratios ± S.E.M. from at least three independent determinations. *p<0.05 or **p<0.01 Vs untreated control or t0, as determined by One-way ANOVA followed by Dunnett’s *post-hoc* test. Kinetics of tPA mRNA expression in untreated cells (Ctrl) or following treatment with 10^−7^M PACAP38 at different times (0, 1, 2, 4, 6, 8, 10, 12, 24 and 48h, respectively) as determined by quantitative real time PCR analyses (**c**). Results are presented as mean fold changes with respect to Ctrl at the same experimental time ± S.E.M. Fold changes of tPA gene expression were obtained after normalization to the endogenous ribosomal protein S18 (housekeeping gene) and then calculated using the comparative ΔCt method. Baseline expression levels of the control group (Ctrl) were set to 1. Experiments were performed three times independently. *p<0.05 or ***p<0.001 vs Ctrl at the same experimental time, as determined by ANOVA followed by Dunnett’s *post-hoc* comparison.

To evaluate whether PACAP38 promoted the expression of tPA both at the mRNA and protein level we conducted three separate sets of experiments. First, through Western blot analyses we evaluated which concentration of PACAP reflected a significant induction of tPA protein expression. Cells were treated with increasing concentrations of the peptide (10^−10^ to 10^−6^ M, respectively) for 24h. Experimental data demonstrated that PACAP38 treatment in a graded manner increased tPA relative protein abundance, reaching statistically significant changes at the 10^−7^M concentration (F_4,14_ = 7.696, **p<0.01 Vs Control, Fig. 1A *)*. This concentration was thus chosen for subsequent evaluations. Of note, similar experiments carried out using the PACAP-homolog vasoactive intestinal peptide (VIP) revealed that the cognate peptide was unable to induce significant increases in tPA expression at nanomolar concentrations, thus showing some significant effects only at concentrations 100- to 1000-fold higher than that used for PACAP (F_6,20_ = 52.46, **p<0.01 Vs Control, see S1 Fig.), indicative that PACAP ability to induce tPA expression is most likely due to binding to the PACAP-preferring PAC_1_ receptor.

To investigate the kinetics of tPA induction at the protein level we carried out immunoblot analyses in cells treated with 10^−7^M PACAP38 at different time points (0, 3, 6, 12, 24 and 48h) (Fig. 1B). Analyses revealed that a significant PACAP-mediated induction of tPA protein expression occurred within 6h after treatment and lasted over the entire timeframe tested (F_5,17_ = 16.41, **p<0.01 Vs t0). Concurrent qPCR experiments were also run to assess the kinetic of tPA mRNA expression in cells exposed to equal concentrations of PACAP38, but using a more restricted time pattern (0, 1, 2, 4, 6, 8, 10, 12, 24 and 48h, respectively). Depicted in Fig. 1C is shown that PACAP stimulation caused a rapid and robust increase of tPA mRNAs already after 2h (F_9,29_ = 32.31, **p<0.01 Vs unstimulated control, ~3.5-fold over controls), which slightly preceded that observed in protein expression analyses, peaked at 4h (**p<0.01 Vs controls, ~5.8-folds) and then progressively declined to reach levels about 2-fold that of controls from 10h and up to 48h (Fig. 1C).

### Distribution of tPA-like immunoreactivity in RT4-D6P2T cells treated with PACAP38, maxadilan, VIP and BDNF

**Fig. 2:**
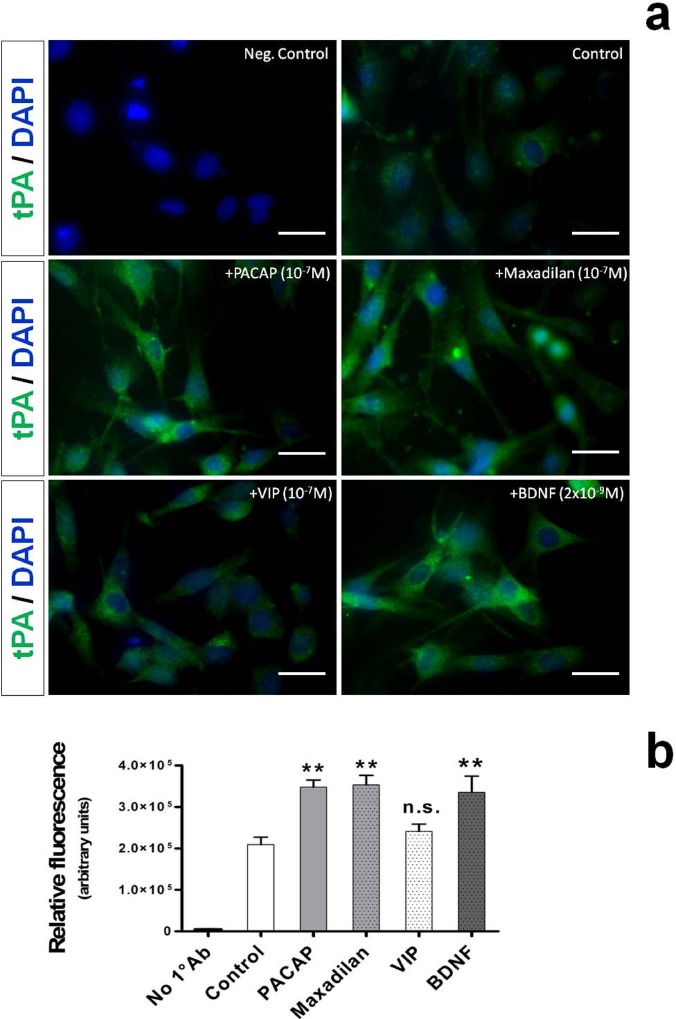
Localization of tPA-like immunoreactivity in RT4-D6P2T cells stimulated with PACAP, maxadilan, VIP or brain-derived neutrophic factor (BDNF). Depicted are representative immunocytofluorescence photomicrographs (**a**) and bar graphs showing semi-quantification of fluorescent intensity (**b**) in cells in which 1° Ab was omitted (Neg. Control), were left untreated (Control) or were treated with the indicated concentrations of PACAP, the PAC_1_ agonist (maxadilan), VIP or BDNF for 24h. Briefly, RT4-D6P2T cells cultured on glass cover slips were fixed in 4% para-formaldehyde, permeabilized with 0.2% Triton X-100, blocked with 0.1% BSA in PBS and then probed with the tPA antibody (1:50 dilution). Detection was then performed using an Alexa fluor 488-conjugated secondary antibody. Nuclei were counterstained with DAPI (#940110 Vector Laboratories). Immunofluorescent images were captured using an Axiovert 40 fluorescence microscope (Carl Zeiss Inc.). Each condition was reproduced in three dishes *per* experiment. Representative photomicrographs were taken from at least three fields *per* dish in a fixed pattern. Original magnification 40X. Scale bar = 30μm.

According to several reports, tPA is sequestered in different intracellular compartments depending on the cell type used [25, 33, 35]. Therefore, we sought to investigate whether tPA had a different localization pattern in the Schwann cell-like culture under study and also if tPA-stimulating agents could affect such a intracellular distribution. To this end, cells were either left untreated or exposed to 10^−7^M PACAP38, an equivalent concentration of maxadilan (a potent PAC_1_ receptor agonist) or VIP and 2 x 10^−9^M BDNF for 24h. In some experiments, we omitted the primary antibody and the relative photomicrograph was used as negative control (Fig. 2A, upper left image).

As evidenced in unstimulated cells, tPA-like immunoreactivity showed sparse cytoplasmic distribution in cell bodies with a more dense punctuate staining along the perinuclear space (Fig. 2A). Indeed, neither stimulation with PACAP38, maxidilan, nor with VIP or BDNF affected tPA-like distribution in RT4-D6P2T cells, although they significantly increased relative fluorescence. Specifically, tPA fluorescent intensity data analyzed using the freely available ImageJ software were consistent with Western Blot and real time RT-PCR data, indicating that PACAP and maxadilan at the same concentration increased tPA reactivity with respect to controls (F_5,29_ = 36.75, **p<0.01 Vs Control, ANOVA followed by Tukey *post-hoc* test) (Fig. 2B). At the concentration tested (10^−7^M) VIP failed to increase tPA reactivity in a significant manner (*p>0.05 Vs Control*), whereas BDNF, a potent neurotrophic factor, efficaciously mimicked PACAP effects on tPA expression (**p<0.01 Vs Control).

### PACAP and BDNF share common signaling pathways to induce tPA expression and activity in RT4-D6P2T cells

**Fig. 3:**
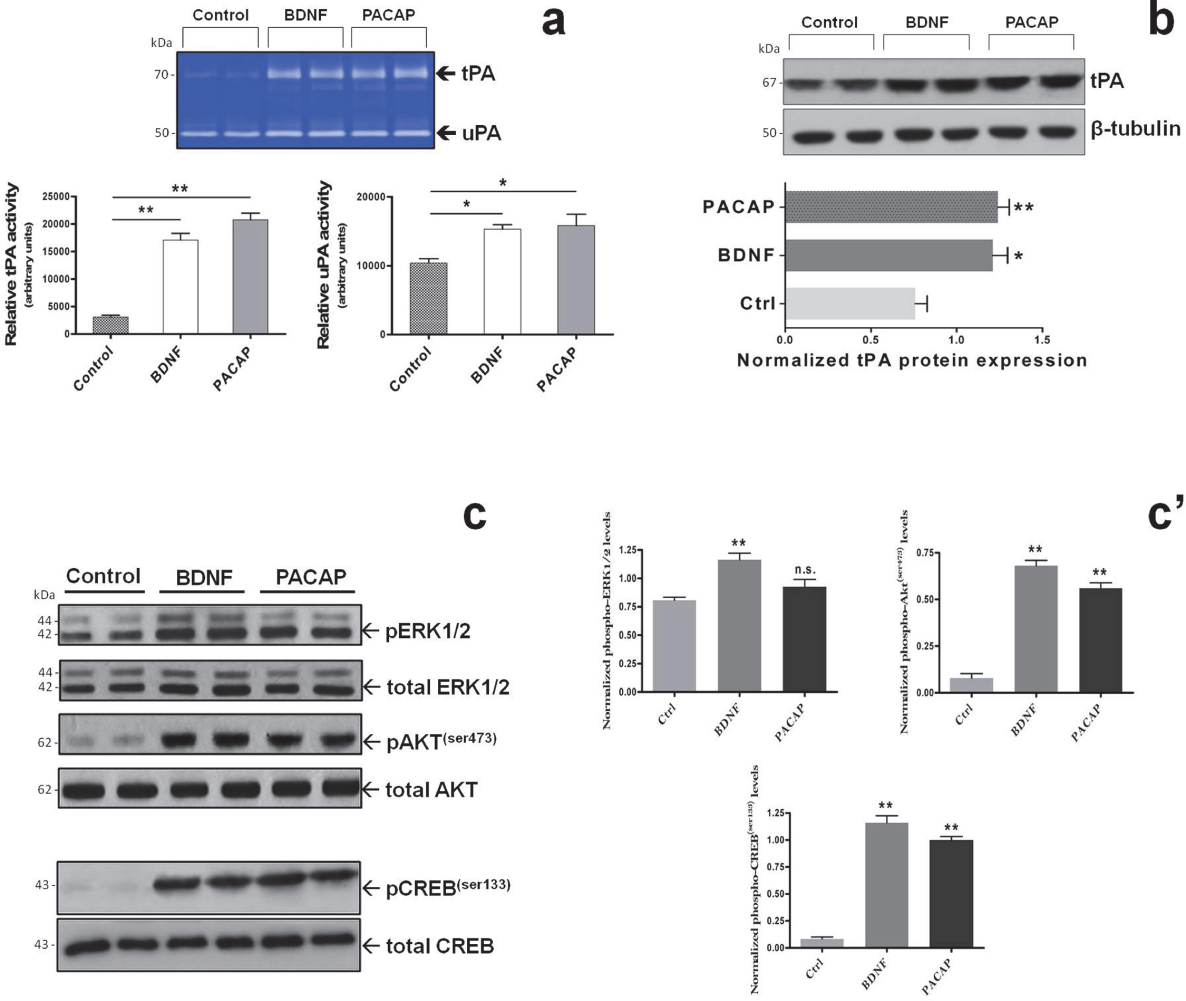
PACAP- and BDNF-mediated induction of tPA expression and activity involves both the MAPK^Erk1/2^ and Akt/cAMP-responsive element binding protein (CREB) signaling pathways. Representative zymographic (**a**) and Western blot analyses (**b**) demonstrating the effects of 10^−7^M PACAP or 2x10^−9^M BDNF treatment on tPA and uPA activity (**a**) and tPA protein expression (**b**) in RT4-D6P2T cells after 24h. tPA and uPA activities were determined by zymography as detailed in MATERIALS AND METHODS. The representative picture shows lytic areas (white bands) on a Coomassie-stained gel (blue background) that correspond to the predicted molecular weight for tPA (64-70kDa) and uPA (~50kDa). Bar graphs depicted below the gel indicate the relative activity of each plasminogen activator, measured as the mean band intensity obtained from at least three separate determinations and calculated using the ImageJ software. *p<0.05 or **p<0.01 Vs Control; ANOVA followed by Dunnett’s *post-hoc* test. (**c** and **c’**) Activation of the MAPK^Erk1/2^ and Akt/CREB signalling cascades was assessed by measuring Erk1/2, Akt and CREB phosphorylation at the indicated phospho-sites using the same procedures described in Fig. 1. Relative activation was calculated by normalizing each phospho-protein level over the corresponding unphosphorylated protein, which also served as loading control. Each plotted result represents the mean ± SEM from three separate experiments. **p<0.01 Vs Control; ANOVA followed by Dunnett’s *post-hoc* test. n.s. = not significant.

Based on the above reported effects showing that both PACAP and BDNF were able to similarly induce tPA expression (Fig. 2), we thought it was compelling to also determine whether both molecules affected tPA proteolytic activity, and to investigate the intracellular signaling pathways involved. To answer the first question, zymographic analyses of tPA activity (and also urokinase PA, namely uPA) were conducted in cells either left untreated (Control) or treated with 2 x 10^−9^M BDNF or 10^−7^M PACAP38 for 24h. Quantification of tPA lytic bands on gels (white bands corresponding to 64-67kDa on a Coomassie-stained background) indicated that BDNF and PACAP similarly increased tPA activity as compared to untreated cells (F_2,11_ = 36.75, **p<0.01 Vs Control, ANOVA followed by Dunnett’s *post-hoc* test) (Fig. 3A). Interestingly enough, uPA activity was also increased in response to BDNF and PACAP treatment, although to a lesser extent than that observed for tPA (F_2,11_ = 7.73, *p<0.05 Vs Control, ANOVA followed by Dunnett’s *post-hoc* test) (Fig. 3A). To validate whether induction of tPA activity by PACAP and BDNF correlated well with induction of protein expression, lysates from cells stimulated as indicated above were also tested for tPA expression by immunoblots. We found that both BDNF and PACAP increased tPA protein expression in stimulated cells with respect to untreated controls with almost equal efficiency (BDNF and PACAP: F_2,8_ = 12.99, *p<0.05 and **p<0.01 Vs Control, respectively; ANOVA followed by Tukey *post-hoc* test) (Fig. 3B). In addition, because tPA gene expression is known to be transcriptionally regulated by the transcription factor cAMP-response element binding protein (CREB) [8, 32] whose activity is mainly regulated by the MAPK and/or Akt signalling cascades, we investigated whether or not BDNF and/or PACAP might act via either intracellular pathway to induce CREB phosphorylation at the serine 133 residue (a priming site necessary to induce CREB transcriptional activity) to induce tPA expression. Western blot data and semiquantification of blots showed that PACAP treatment did not affect Erk1-2 phosphorylation in stimulated cells (*F_2,8_ = 12.15*, *p>0.05 Vs Control*, not significant; ANOVA followed by Dunnett’s *post-hoc* test) whereas treatment with BDNF (2x10^−9^M) markedly increased phospho-Erk1-2 levels (**p<0.01 Vs Control) (Fig. 3C and 3C’). Of interest, PACAP and BDNF both potently induced Akt phosphorylation at serine 473 (*F_2,8_ = 137.5*, **p<0.01 Vs Control) (the Akt catalytic site activated by phosphatidylinositol-4,5-bisphosphate 3-kinase), a finding that might be related to the Akt-mediated CREB phosphorylation at Ser133 in cells stimulated with BDNF (*F_2,8_ = 173.5*, **p<0.01 Vs Control) and PACAP (**p<0.01 Vs Control) (Fig. 3C and 3C’).

### Activation of the Akt signaling pathway by PACAP but not BDNF is both sufficient and necessary for tPA induction in RT4-D6P2T cells

**Fig. 4:**
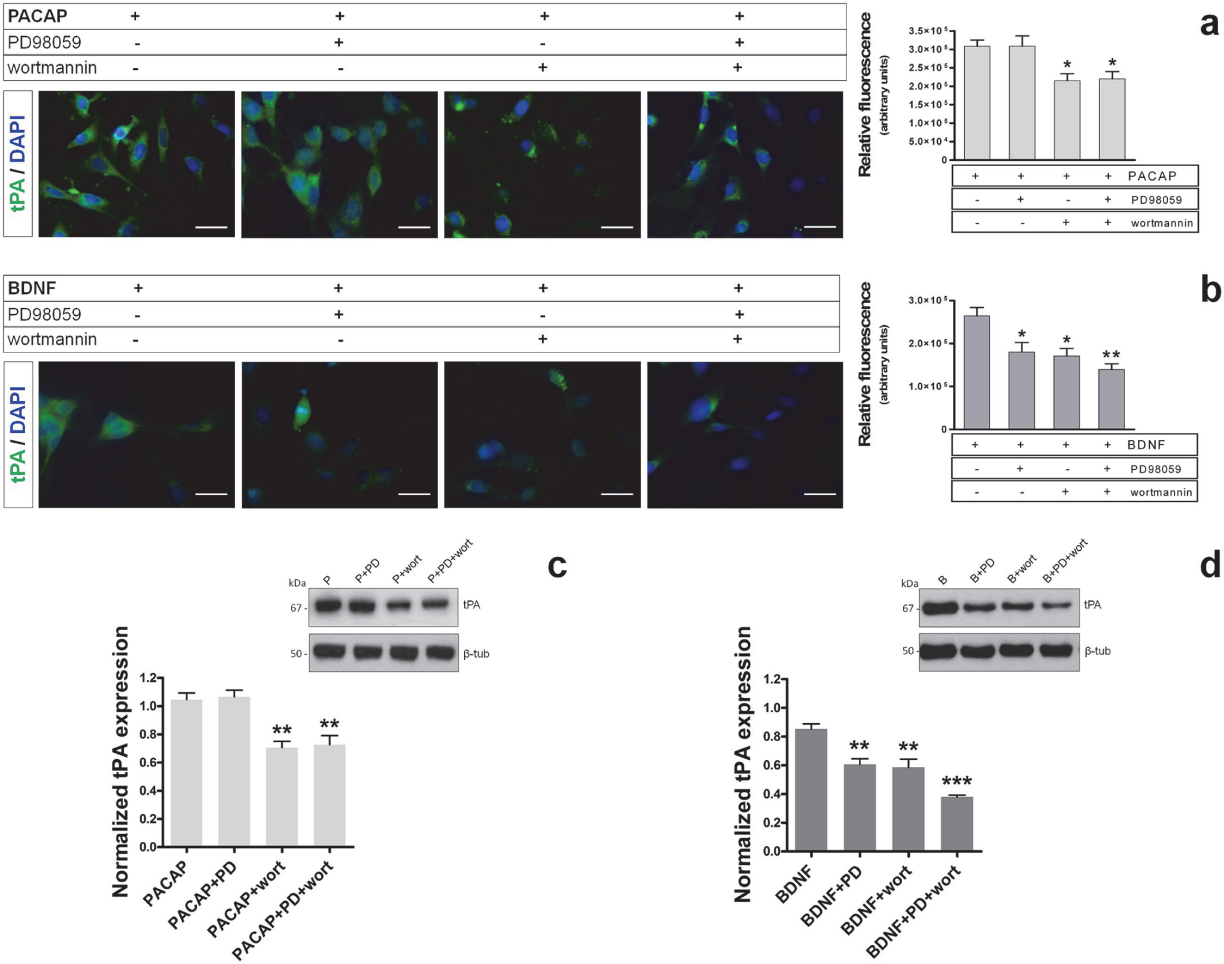
Effects of MAPK^Erk1/2^ and PI3K inhibitors on tPA expression in RT4-D6P2T cells. Photomicrographs and relative quantifications of tPA-like immunoreactivity in RT4-D6P2T cells that were pretreated for 30 min with either 50μM PD98059 (a MEK1 inhibitor), 10μM wortmannin (a PI3K inhibitor) or a combination of both compounds and exposed to PACAP (**a**) or BDNF (**b**) for 24h. Immunofluorescent images were captured using an Axiovert 40 fluorescence microscope (Carl Zeiss Inc.). Each condition was reproduced in three dishes *per* experiment. Representative photomicrographs were taken from at least three fields *per* dish in a fixed pattern. Original magnification 40X. Scale bar = 30μm. For more details on the procedure please refer to MATERIALS AND METHODS section. Bar graph data is presented as the mean ± SEM. *p<0.05 Vs Control; ANOVA followed by Tukey *post-hoc* test. (**c** and **d**) Representative results from parallel immunoblot experiments showing the effect of pathway inhibitors on PACAP- or BDNF-mediated induction of tPA expression. Normalized expression levels were calculated using β-tubulin as the loading control. Each result displayed in the two bar graphs represents the mean ± SEM from three independent assessments (**c** and **d**, respectively). **p<0.01 Vs Control; ANOVA followed by Dunnett’s *post-hoc* test.

Consistent with the activation/phosphorylation of Akt observed in PACAP or BDNF-treated cells and MAPK^Erk1/2^ phosphorylation in BDNF-treated cells only, we attempted to establish the effective contribution of each of the two signaling pathways in inducing tPA expression. The concentrations for each pathway inhibitor were selected based on results obtained in our previous study using this same cell line [11]. Immunofluorescence analyses showed that pretreatment (30min) of cells with the MEK1 inhibitor (PD98059, 50μM) did not abrogate PACAP-induced tPA-like immunoreactivity in RT4-D6P2T cells (*F_3,19_ = 6.151*, *p>0.05 Vs Control*, not significant) (Fig. 4A), whereas it completely averted BDNF-mediated effects (*F_3,19_ = 8.396*, *p<0.05 Vs Control) (Fig. 4B). Conversely, pretreatment with the PI3K inhibitor wortmannin (10μM) efficaciously prevented the induction of tPA both by PACAP or BDNF (*p<0.05 Vs Control, respectively) (Fig. 4A and 4B). Interestingly, co-application of both PD98059 and wortmannin did not exert additional inhibitory effects on tPA-like signal with respect to wortmannin only in PACAP stimulated cells (*p>0.05 Vs PACAP+wortmannin treated-cells*). Conversely, pretreatment with both pathway inhibitors acted synergistically to further attenuate tPA-like immunoreactivity in BDNF stimulated cells (**p<0.01 Vs Control, *p<0.05 Vs BDNF+wortmannin treated-cells). These data were further confirmed by Western blot analyses and subsequent quantifications, hence providing even more robust results (wortmannin in PACAP-treated cells: *F_3,11_ = 15.05*, **p<0.01 Vs Control; PD98059+wortmannin in PACAP-treated cells: **p<0.01 Vs Control; wortmannin in BDNF-treated cells: *F_3,11_ = 25.31*, **p<0.01 Vs Control; PD98059+wortmannin in BDNF-treated cells: ***p<0.001 Vs Control) (Fig. 4C and 4D). These findings suggested that in PACAP-stimulated cells activation of the Akt signalling cascade is necessary for the induction of tPA expression, whereas in BDNF-stimulated cells concurrent activation of Akt, MAPK^Erk1/2^ and/or other signaling seem to be required.

### BDNF-driven induction of tPA expression involves a potentiated endogenous PACAP/PAC_1_ receptor signaling

**Fig. 5:**
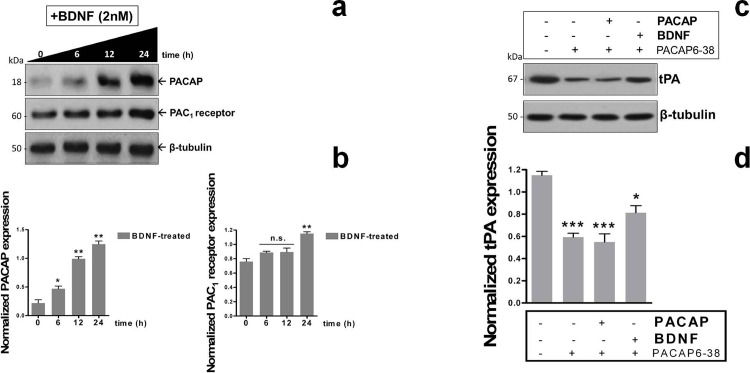
BDNF boosts endogenous PACAP/PAC_1_ receptor signaling to induce tPA expression in RT4-D6P2T cells. Western blots and related quantifications (**a** and **b**) showing the effect of 2x10^−9^M BDNF treatment on PACAP peptide and PAC_1_ receptor expression at different exposure times (0, 6, 12 and 24h). For experimental procedures please refer to the appropriate section in MATERIALS AND METHODS. Results in (**b**) are the mean ± SEM of normalized values obtained from at least three separate experiments. β-tubulin was used as the loading control. *p<0.05 or **p<0.01 Vs Control; ANOVA followed by Tukey *post-hoc* test. Inhibitory effects of the non-specific PAC_1_/VPAC type receptor antagonist PACAP6-38 (10μM) on tPA protein expression were determined by Western blots (**c**). Cells were either left untreated (control) or pretreated with PACAP6-38 in the absence or presence of PACAP or BDNF and tPA expression was evaluated. Quantification of bands was performed by normalizing tPA band density to that of β-tubulin, which was used as loading control. Each experiment was reproduced three times with similar results. Depicted in the bar graph are the average ratios ± SEM. *p<0.05 or ***p<0.001 Vs Control; ANOVA followed by Tukey *post-hoc* test.

Given that BDNF was capable to induce a similar induction of tPA expression as observed in cells stimulated with PACAP, we suspected that BDNF-mediated effects might be due to an increase in the endogenous PACAP/PAC_1_ signaling system. Therefore, we first assessed whether BDNF stimulated the endogenous expression of PACAP and of the PAC_1_ receptor in this cell line and then evaluated if pretreatment with a PAC1/VPAC2 receptor antagonist could interfere with BDNF stimulatory effects on tPA. Indeed, we discovered that supplementation of BDNF to cells (2x10^−9^M) at different time points (0, 6, 12 and 24h, respectively) progressively increased PACAP protein expression (*F_3,11_ = 90.3*, at 6h → *p<0.05 Vs t0, at 12 and 24h → **p<0.01 Vs t0; ANOVA followed by Dunnett’s *post-hoc* test) (Fig. 5A and 5B). BDNF-mediated induction of PAC_1_ receptor expression showed a clear trend towards an increase, but statistical significance was reached only after 24h treatment (*F_3,11_ = 18.6*, *at 6h and 12h → p>0.05 Vs t0*, at 24h → **p<0.01 Vs t0) (Fig. 5A and 5B).

Parallel experiments using the PACAP/VIP receptor antagonist PACAP6-38 demonstrated that at the concentration used (10μM) [10, 11] the receptor antagonist completely blocked exogenous PACAP-stimulated tPA expression (*F_3,11_ = 25.03*, ***p<0.001 Vs Control, ANOVA followed by Tukey *post-hoc* test) but also significantly reduced tPA levels in cells that were not stimulated (***p<0.001 Vs Control) (Fig. 5C and 5D), suggesting that the endogenous PACAP system is constitutively active in this cell line. In BDNF-stimulated cells, PACAP6-38 only partially impeded tPA induction (*p<0.05 Vs Control), inferring on the possibility that BDNF might in part sustain tPA expression through alternative mechanisms.

### Serum starvation induces tPA expression in RT4-D6P2T cells

**Fig. 6:**
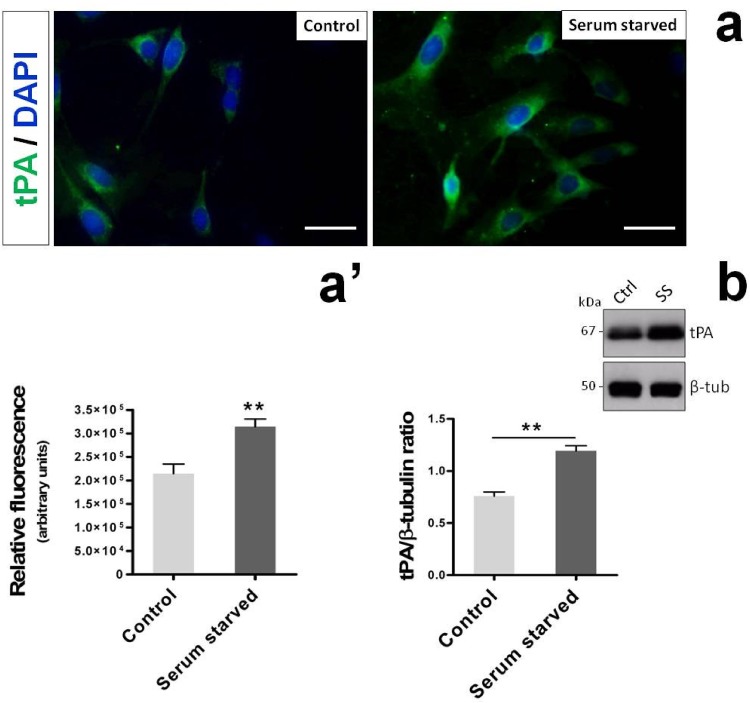
Serum withdrawal induces tPA expression in RT4-D6P2T cells. Photomicrographs (**a**) and relative quantifications of tPA-like immunoreactivity in RT4-D6P2T cells (**a’**) cultured either in the presence (Control) or absence of serum (Serum Starved) after 24h. Immunofluorescent images were captured using an Axiovert 40 fluorescence microscope (Carl Zeiss Inc.). Each condition was reproduced using at least three dishes *per* experiment. Representative photomicrographs were taken from at least three fields *per* dish in a fixed pattern. Original magnification 40X. Scale bar = 30μm. Details on the procedure can be found in MATERIALS AND METHODS section. (**a’**) Bar graph depicts the relative fluorescence ± SEM, expressed as arbitrary units. **p<0.01 Vs Control, as determined using the unpaired two-tailed Student *t*-test. (**b**) Representative immunoblot and related quantifications of bands obtained by resolving cell lysates from cultures treated as indicated above. Normalized tPA expression levels were calculated using β-tubulin as the loading control. Each result displayed in the bar graph represents the mean ± SEM from three independent experiments. **p<0.01 Vs Control; Unpaired two-tailed Student *t*-test. Ctrl = 10% FBS cultured RT4-D6P2T cells after 24h. SS = Serum starved cells.

Recently we have demonstrated that RT4-D6P2T cells undergoing serum starvation show an increased endogenous expression of both PACAP and its high affinity receptor PAC_1_ [11]. In line with these studies and with current findings suggesting that a boosted endogeonous PACAP/PAC_1_ system might be involved in upregulating tPA expression in this cell line, we sought to investigate whether or not a brief period of serum withdrawal was also capable to induce tPA expression. To test this hypothesis, RT4-D6P2T cells were cultured either in the presence of 10% FBS (Control) or in total absence of serum (Serum Starved) for 24h. Afterwards, tPA expression in both groups was assessed both by immunofluorescence and immunoblot analyses. As apparent from Fig. 6A and 6A’, serum depletion over a period of 24h was sufficient to significantly increase tPA immunosignal in comparison to normally fed cells (t_8_ = 3.823, **p<0.01 Vs Control; Unpaired two-tailed Student t-test). Such result was confirmed by Western blot analyses carried out on lysates obtained under the same experimental settings (Fig. 6B). Indeed, tPA protein expression levels increased at statistically significant levels after 24h serum starvation (t_4_ = 6.989, **p<0.01 Vs Control), further supporting the idea that an heightened PACAP/PAC_1_ signaling might be responsible, at least in part, for the increased tPA expression in the RT4-D6P2T Schwann cell-like culture.

## Discussion

In the present study we have provided evidence that PACAP, through its interaction with the high affinity PAC_1_ receptor, stimulates tPA expression and proteolytic activity in RT4-D6P2T Schwann-cell like cultures. Throughout this study we were also able to identify the Akt/CREB intracellular cascade as the main trigger of the transcriptional machinery that drives tPA expression. Finally, we further demonstrated that brain-derived neutrophic factor (BDNF), a trophic molecule recently found to induce PACAP expression in rat sensory and motor neurons after nerve injury [34] is likewise able to increase the expression of endogenous PACAP, PAC_1_ receptor and in turn of tPA in our cell system, likely acting as a booster of the endogenous PACAP/PAC_1_ signaling system.

Over the years, PACAP has been designated has a potent neuroprotective molecule, able to exert several biological functions in different cell types [11, 39, 44], including the one used in the present work [9, 10, 12]. PACAP and its receptors are normally expressed in peripheral nerves (sciatic nerve) and their levels are increased following axotomy [49] or nerve compression [34]. Through other studies it was also shown, though indirectly, that dissociated cultures from monkey trigeminal nerves contain Schwann cells that express functional PAC_1_ receptors [31]. This and other studies [26] have set the stage to proceed with further investigations on the role of this neuropeptide in relationship to Schwann cells biology and beyond.

Indeed, further biological functions for PACAP have emerged so far and are still arising, including immunomodulatory [1, 41–47], and regenerative functions both in the CNS and PNS [5–7, 22, 35]. These beneficial functions of PACAP suggest its potential therapeutic application for the treatment of autoimmune diseases, neuroinflammatory disorders and perhaps to ameliorate nerve repair after injury. In either case, understanding the mechanisms involved in PACAP regenerative functions in the nervous system and in response to different challenges (i.e. injury, oxidative stress, excessive cytokine release etc.) has remained a complex task, since the peptide acts in a cell/tissue-specific manner and through the activation of a plethora of signaling pathways. In this study we focused our attention in understanding whether PACAP induced tPA expression and proteolytic activity in the RT4-D6P2T Schwann cell-like culture. We took advantage of this cell line because it has demonstrated to be a suitable tool to study Schwann cell behavior, and it shares several biochemical and structural properties with primary Schwann cells [10, 11, 21, 37]. The rationale behind this study was based on previous observations suggesting that exogenous administration of PACAP promoted the expression and activity of tPA in neurons [35], but also on findings from independent research groups showing that both PACAP and tPA expression levels, secretion and activities are increased at the site of injury after peripheral nerve damage [2, 40, 51].

tPA is a well-known proteolytic enzyme belonging to the family of plasminogen activators and is endowed with a myriad of biological functions. The first evidence indicating that Schwann cells are an important source of plasminogen activators (including tPA) was published in 1984 [24], followed by another study carried out seven years later indicating that neurons regulate Schwann cell release of both tPA and urokinase PA (uPA) [14]. A subsequent research conducted by Akassoglou and coworkers demonstrated that tPA secreted from Schwann cells acted as a proteolytic enzyme necessary to degrade fibrin and to promote axonal regeneration and remyelination after sciatic nerve injury [2]. Later, the same research group discovered that fibrin was the main responsible for the inhibition of remyelination during peripheral nerve injury since its accumulation at the injury site blocks Schwann cell differentiation [3] and migration [4]. As initially hypothesized, in the course of our study we found that PACAP, but not its structurally-related homolog VIP, acted as a potent inducer of tPA expression and activity in this cell line (Fig. 1–3). In line with these findings, following application of PACAP, the kinetics of tPA mRNA showed a rapid increase, with a peak after 4h. This result, almost overlapping with that observed after PACAP stimulation in a neuronal cell line (6h) [35], may imply that upon nerve injury, Schwann cells are rapidly recruited through the axonal release of PACAP (and maybe other factors) by affected neurons to aid in the digestion of cellular debris, to protect axons from degradation and finally to promote nerve remyelination. Alternatively, it is also possible that, in response to injury, myelinating glia regulates its own fate and proteolytic activity *via* the production and release of PACAP, possibly through an autocrine/paracrine loop. However, these hypotheses still need to be confirmed.

Regarding the different responsiveness of RT4-D6P2T cells to PACAP and VIP ability to induce tPA, considering the equal affinity of VPAC type receptors for both PACAP and VIP and the lack of activity of VIP on tPA expression (except at micromolar concentrations → see S1 Fig.) these result suggested a major involvement of the PACAP-preferring PAC_1_ receptor, in agreement with previous reports indicating that PAC_1_ receptors are also elevated following peripheral nerve injuries [40].

Once ascertained that PACAP was an effective inducer of tPA mRNA and protein expression we also attempted to determine if proteolytic activity was also increased. Furthermore we tested whether BDNF, a trophic factor with demonstrated efficacy in peripheral nerve regeneration after injury (recently reviewed by [36]) and whose functional binding receptors for BDNF (p75NTR, TrkB) have been well-characterized in Schwann cells ensheating peripheral nerves [13, 19, 48] could mimic PACAP stimulating effects on tPA expression and activity. We discovered that PACAP significantly increased tPA activity and, to a minor extent, uPA activity on its substrate plasminogen (Fig. 3A). BDNF was equally potent to induce tPA expression and proteolytic activity in Schwann cell-like cultures. Investigations on the signaling pathways indicated that BDNF acted both through the MAPK^Erk1/2^ and the PI3K/Akt signaling pathways whereas PACAP activated the latter only (Fig. 3C and 3C’), consistent with recent findings from our laboratory [11]. Despite the differences, both PACAP and BDNF similarly activated cAMP-responsive element binding protein (CREB), the main transcriptional activator of the tPA gene [8, 32]. Pharmacological inhibition of the MAPK^Erk1/2^ signaling pathway had no effect on PACAP-induced tPA expression but completely prevented BDNF effects. Similarly, wortmannin, a PI3K inhibitor, prevented the effects of both trophic molecules, suggesting that this signaling pathway is both necessary and sufficient to increase tPA, but only in PACAP-treated cells. Further analyses uncovered more complex functions associated to BDNF. Indeed, exogenous treatment with the neurotrophic agent strikingly increased both PACAP and PAC_1_ receptor expression levels (Fig. 5A and 5B), in line with recent observations by Pettersson and coworkers (2014) [34] demonstrating that endogenous BDNF is an inducer of PACAP expression in injured nerves. This result prompted us to investigate whether antagonizing PAC_1_/VPAC type receptors hampered, at least in part, not only PACAP stimulating effect, but also the one associated to BDNF. As expected, we found that PACAP6-38, a non-specific PAC_1_/VPAC receptor antagonist, showed intrinsic inhibitory activity in cells. In particular, tPA expression levels were similarly downregulated in unstimulated and PACAP-stimulated cells (Fig. 5C and 5D), suggesting that the endogenous PACAP/PAC_1_ signaling is constitutively active and contributes to maintain tPA levels under basal conditions. tPA levels were also dampened in BDNF-stimulated cells, although to a minor extent than for PACAP. We hypothesize that the partial reduction obtained in BDNF-treated cells could account for the activation of alternative intracellular pathways, including the MAPK^Erk1/2^ signaling previously observed (Fig. 3B). Finally, to further confirm the specific involvement of the heightened PACAP/PAC_1_ signaling in tPA induction, enzyme expression levels were assessed in RT4-D6P2T cells undergoing a brief period of serum starvation (24h), a condition known to trigger the expression of PACAP and its related receptors in this cell line [11]. Results confirmed that tPA expression was increased as a consequence of serum starvation (Fig. 6), thus providing a further evidence supporting the role of the PACAP/PAC_1_ system in tPA induction.

## Conclusions

In conclusion, the findings obtained in the present study helped us to identify PACAP as a potent inducer of tPA expression and activity in Schwann cell-like cultures and allowed to characterize the signaling pathways involved. In addition, our studies using BDNF suggest that the trophic molecule, released from Schwann cells, may act as an upstream regulator of the endogenous PACAP/PAC_1_ system to promote proteolytic activity and facilitate nerve recovery after injury.

## Supporting Information

S1 Fig Dose-dependent effects of VIP on tissue plasminogen activator (tPA) protein expression. Depicted in the figure are representative immunoblots and related densitometry showing the absence of significant effects on tPA protein expression after treatment of cells with increasing concentrations of VIP (10^−10^M to 10^−6^M, respectively) after 24h. A significant induction of tPA expression was observed only at highest concentrations tested (10^−5^M and 10^−4^M VIP, respectively), suggesting a major involvement of PAC_1_ receptors. Protein extracts (20μg) obtained from rat RT4-D6P2T cell lysates were separated by SDS-PAGE and transferred to nitrocellulose membranes. Afterwards, membranes were incubated using a rabbit anti-tPA (1:300, sc-15346, Santa Cruz Biotechnology) and a rabbit anti-β-tubulin antibody (H-235, cat n. sc-9104, Santa Cruz Biotechnology; 1:500) and scanned with an Odyssey Infrared Imaging System, as described in Materials and Methods section. Densitometric analyses were performed using the ImageJ software and values obtained were normalized to β-tubulin, which was used as loading control. Results are expressed as the average ratios ± S.E.M. from three independent determinations. **p<0.01 Vs untreated controls, as determined by One-way ANOVA followed by Dunnett’s *post-hoc* test.
(TIF)
