# Supplementary figures and images for: PACAP Interacts with PAC1 Receptors to Induce Tissue Plasminogen Activator (tPA) Expression and Activity in Schwann Cell-Like Cultures
Source: PLoS One. 2015 Feb 6;10(2):e0117799. doi: 10.1371/journal.pone.0117799 (PMC4319891; doi:10.1371/journal.pone.0117799)

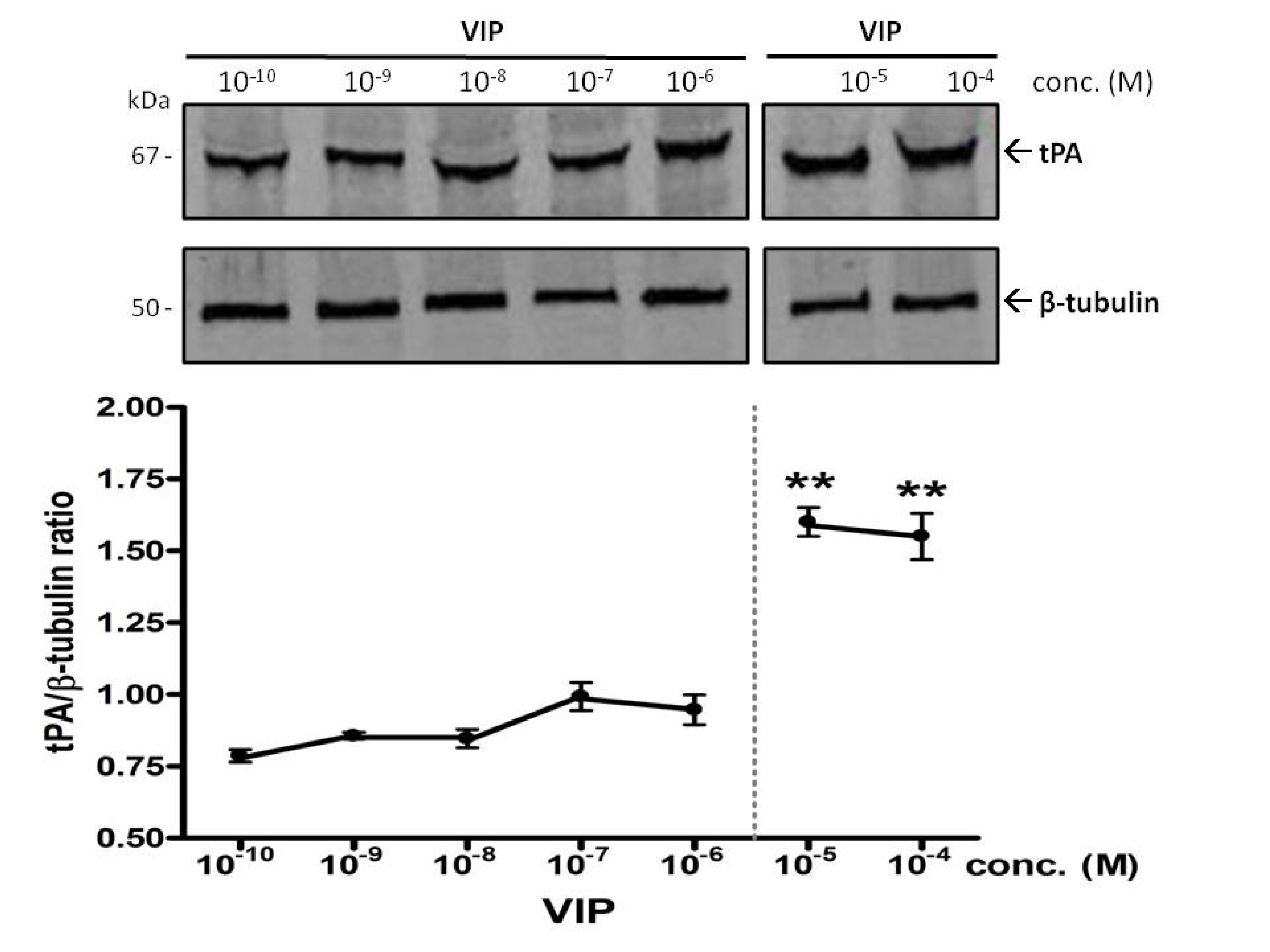

Supplement: S1 Fig — Depicted in the figure are representative immunoblots and related densitometry showing the absence of significant effects on tPA protein expression after treatment of cells with increasing concentrations of VIP (10−10M to 10−6M, respectively) after 24h. A significant induction of tPA expression was observed only at highest concentrations tested (10−5M and 10−4M VIP, respectively), suggesting a major involvement of PAC1 receptors. Protein extracts (20μg) obtained from rat RT4-D6P2T cell lysates were separated by SDS-PAGE and transferred to nitrocellulose membranes. Afterwards, membranes were incubated using a rabbit anti-tPA (1:300, sc-15346, Santa Cruz Biotechnology) and a rabbit anti-β-tubulin antibody (H-235, cat n. sc-9104, Santa Cruz Biotechnology; 1:500) and scanned with an Odyssey Infrared Imaging System, as described in Materials and Methods section. Densitometric analyses were performed using the ImageJ software and values obtained were normalized to β-tubulin, which was used as loading control. Results are expressed as the average ratios ± S.E.M. from three independent determinations. **p<0.01 Vs untreated controls, as determined by One-way ANOVA followed by Dunnett’s post-hoc test. (TIF) [file pone.0117799.s001.tif]
